# Foreign Body Ingestion by a Psychiatric Patient Requiring an Appendectomy: A Case Report

**DOI:** 10.7759/cureus.46977

**Published:** 2023-10-13

**Authors:** Luisa F Cuervo-Ollervides, José L Serafio-Gómez, Illich A Jauregui-Salazar, Carlos R Cervantes-Sánchez

**Affiliations:** 1 General Surgery, Chihuahua City General Hospital “Dr. Salvador Zubirán Anchondo”, Chihuahua, MEX; 2 Educational Research Department, Faculty of Medicine and Biomedical Sciences, Autonomous University of Chihuahua, Chihuahua, MEX; 3 General Surgery, Chihuahua City General Hospital "Dr. Salvador Zubirán Anchondo", Chihuahua, MEX

**Keywords:** psychiatric disorder, needle, foreign body, appendectomy, appendicitis

## Abstract

In the surgical field, the correct approach to the psychiatric patient represents a medical challenge, given the special considerations to be taken in the individualization of their diagnosis and treatment. We present an uncommon case of a 29-year-old patient with associated psychiatric pathology who presented to the emergency room after the introduction of two foreign bodies into the nasal cavity. After the endoscopic removal of one of the foreign bodies, the X-ray follow-up shows a second foreign body into the esophagus, which progressed to the vermiform appendix, causing the classical clinical signs of acute appendicitis as a complication. The importance of considering that events treated by the otorhinolaryngology area may have complications for urgent management by the general surgery service is denoted in this article.

## Introduction

Foreign body ingestion is a complex clinical picture, most commonly presented in the pediatric and psychiatric population [1]. However, less than 1% are estimated to require surgical intervention [2]. Cases of appendicitis have been reported due to the ingestion of projectiles, stones, fish bones, piercings, razor blades, teeth, batteries, and plant matter, with association to granulomas, perforation, adhesions, and infection [1-6].

The transit of foreign bodies through the digestive tract implies the passage through tract narrowing, sphincters, and tract angulations; propulsion through forced peristaltic movements; mixing with the rest of the food bolus and feces; and, of course, avoiding being impacted in blind bags such as the appendix. Failure in any step can bring complications, such as impaction, perforation, and appendicitis, which may or may not be symptomatic [7].

## Case presentation

A 29-year-old female attended the emergency room assisted by her caregiver. It was referred to a clinical picture from four days prior to her admission when a needle was inserted into her left nostril and a stone into her right nostril, which later caused nasal pain, light epistaxis, and rhinorrhea. Additionally, she has been treated for schizophrenia since age 14 with a previous history of removal of foreign bodies from the nose and ear canal on several occasions.

**Figure 1:**
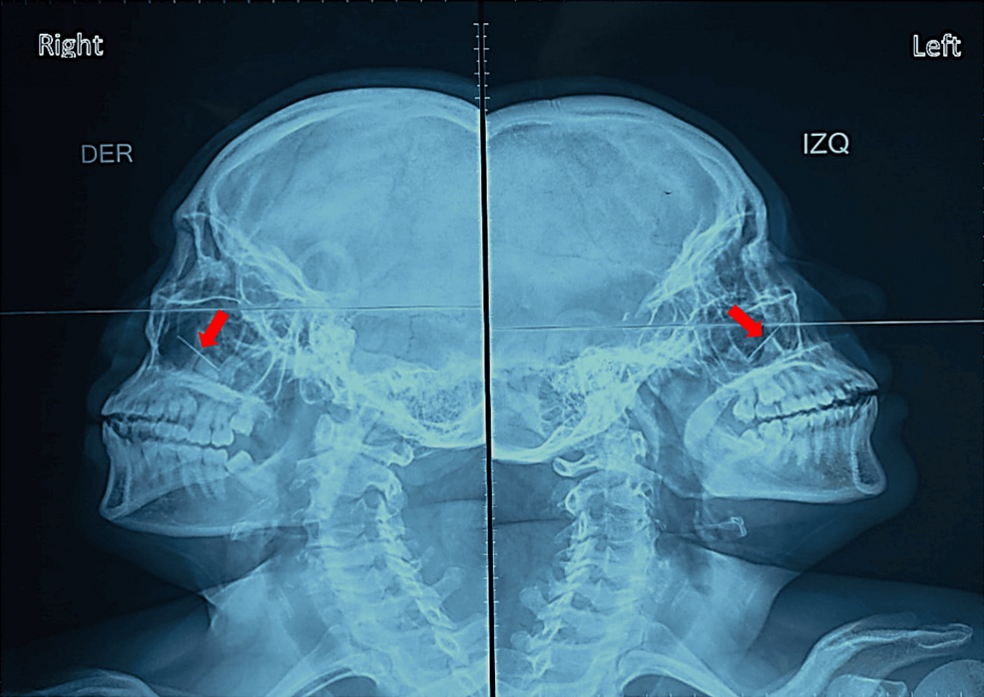
Skull X-ray Admission skull X-ray of both sides (red arrow points to needle)

The patient appears anxious and uncooperative, with light epistaxis. A skull X-ray showed a radiopaque object in the left nostril, approximately 3 cm × 1 mm (Figure 1).

An attempt was made to remove the foreign body in the emergency department without success, so the otorhinolaryngology service scheduled the patient for endoscopic removal. A stone was removed from the right nostril. However, portable X-rays showed that the left nostril foreign body had moved to the thoracic esophagus.

**Figure 2:**
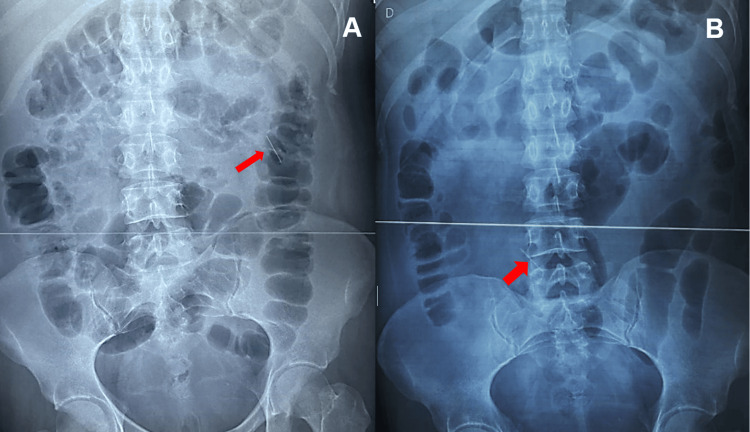
Abdominal X-ray Progression of the metallic needle in the gut (A) 12 hours after admission (B) 48 hours after admission

The patient was kept under observation, and an endoscopy was scheduled, in which no esophageal foreign body was observed. An abdominal X-ray showed that the needle already passed the stomach into the small gut and beyond after 48 hours (Figure 2).

Meanwhile, the psychiatric department adjusted her antipsychotic treatment and follow-up upon hospital discharge.

**Figure 3:**
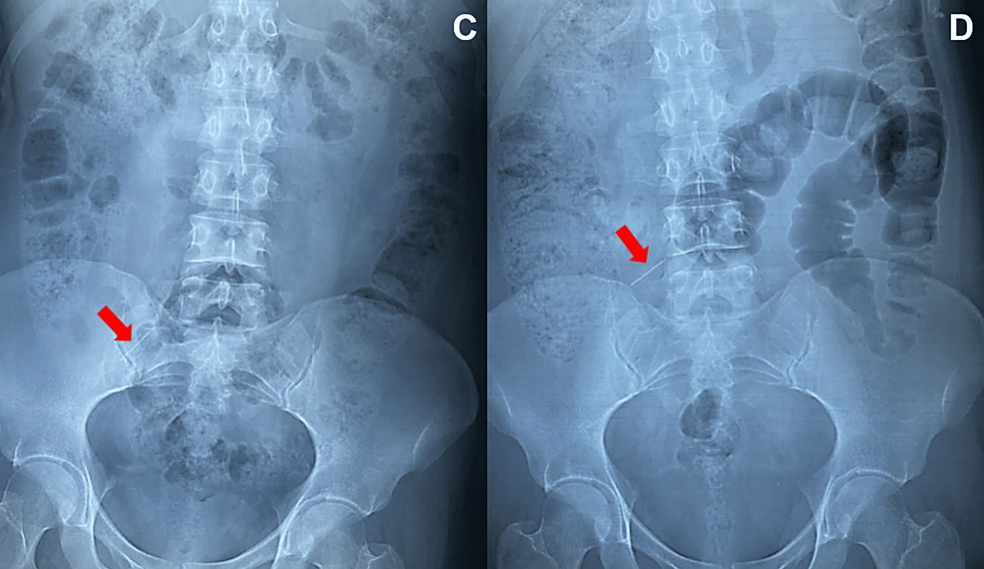
Abdominal X-ray Progression of the metallic needle to the ileocecal valve area (no further progression with a 24-hour difference in time between C and D)

Six days after her admission, the patient started with light and vague abdominal pain, presenting at the physical exploration of classical appendicitis clinical signs (McBurney, Psoas, Obturator, Rovsing, Lanz, right Capurro, and Dunphy). The abdominal X-ray confirms that the metallic, sharp object has been progressing into the gut (Figure 3).

Blood studies reported leukocytes of 5.5 K/uL; hemoglobin, 11.5 g/dL; hematocrit, 34.8%; platelets, 206,000; lymphocytes, 25.5%; and neutrophils, 67.6%.

**Figure 4:**
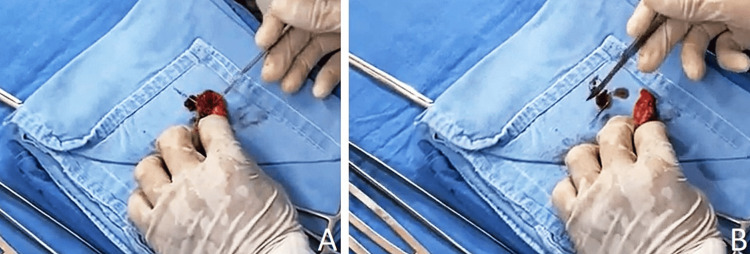
Vermiform appendix Transoperative extraction of the needle from the appendix lumen

An urgent exploratory laparotomy was performed in search of the foreign object, which was finally found in the lumen of the appendix (Figure 4).

An appendectomy was performed, finding an edematous appendix of 7 × 1 × 1 cm with a metal needle inside its lumen, and the sample was sent to the pathology department for final diagnosis.

In the postoperative period, the patient was stable, cooperative, and asymptomatic, tolerating the oral route, with vital signs within normal values, and had no complications until discharge with a follow-up appointment in three weeks.

The histopathological study was reported three days later, with a congestive mesoappendix measuring 1.3 cm and incipient acute appendicitis measuring 10 × 0.8 cm.

## Discussion

In patients without congenital anomalies of the digestive tract, it is known that objects smaller than 2.5 × 6 cm, having passed the esophagus, are expected to be spontaneously expelled in less than a week. Foreign body appendicitis is rare, with incidences hovering around one in 20,000 [1,8]. Less than 0.0005% of ingested foreign bodies are estimated to be retained on suspicion [6,8-10]. The expected complications depend on the constitution of the foreign body and its way of interacting with the medium in which it is housed.

Object characteristics predispose to impaction in certain areas. Small objects with rounded edges tend to stop in the esophageal narrowing, while the elongated ones on the pylorus and the pointed and/or sharp ones tend to stop their transit in angled places such as the duodenum, duodenojejunal junction, ileocecal valve, and vermiform appendix. Foreign objects entering the appendix have been associated with relatively low motility of the cecum and small size of the orifice to the appendicular lumen [2,3,7].

Appendiceal symptoms are a red focus in the clinical evaluation after the ingestion of objects; however, linking foreign body ingestion with appendiceal symptoms is usually difficult, especially in psychiatric patients with poor self-care or lack of control of their underlying pathology. Likewise, asymptomatic patients may even have forgotten the ingestion by not giving importance to it, missing an important pillar in the presumptive etiological diagnosis [4,11,12]. It has been reported that up to 29% of retained appendiceal foreign bodies are asymptomatic [11]. In the clinical case presented here, poorly controlled psychiatric pathology was denoted as a risk factor.

The ingestion of foreign bodies is less common in adults than in children. It is seen more frequently in inmates and those with a previous psychiatric diagnosis with a history of self-injury, which being 89% of those admitted to the emergency room [13].

Surveillance through serial radiographs is advantageous for detecting a failure in transit through the intestines, although its use must be individualized. The use of USG, for example, is recommended to avoid radiation exposure in pediatrics from both follow-up and first-time studies in the diagnostic approach [6]. The predilection for X-rays is based on their availability, low cost, image fidelity, low amount of radiation compared to CT, and not having the interpretative variability of USG [9]. Likewise, the disadvantage is that the exact location cannot be determined, as with USG and CT [6,14,15]. When transit failure is detected, a prophylactic appendectomy can even be considered in asymptomatic patients [9,10]. This consideration is based on the fact that 75% of retained objects in the appendix are at high risk of complications, which are sharp and pointed [9]. In addition, treating uncomplicated appendicitis conservatively with antibiotic therapy is not a recommended option since up to 95.4% of patients end up still requiring surgical intervention due to the complications developed [16-20]. Another point in favor of appendectomy occurs in the context of a failed endoscopic removal [8].

Underestimating the probability of complications is more common, having verified that the foreign body has passed through the esophagus, as up to 93% are expected to be expelled without major problems [11]. Even with this, we must not forget the education of the patient or their caregivers for the identification of certain symptoms in ambulatory care for the monitoring of uncomplicated pictures.

## Conclusions

The ingestion of foreign bodies constitutes a common entity in the emergency consultation. Even with a good prognosis after having passed through the intestines, the possibility of complications should not be minimized. The conduct to follow will depend on the characteristics of the object, its quantity, location, and the context of the patient, individualizing every case. The surgical approach continues to be the most successful option and is recommended even prophylactically in asymptomatic patients with foreign body retention in the appendix.
